# Electrospun Bimetallic NiCr Nanoparticles@Carbon Nanofibers as an Efficient Catalyst for Hydrogen Generation from Ammonia Borane

**DOI:** 10.3390/nano9081082

**Published:** 2019-07-28

**Authors:** Robert M. Brooks, Ibrahim M. Maafa, Abdullah M. Al-Enizi, M. M. El-Halwany, Mohd Ubaidullah, Ayman Yousef

**Affiliations:** 1 Department of Civil and Environmental Engineering, Temple University, 1947 N. 12th Street, Philadelphia, PA 19122, USA; 2 Department of Chemical Engineering, Faculty of Engineering, Jazan University, Jazan 45142, Saudi Arabia; 3 Department of Chemistry, College of Science, King Saud University, Riyadh 11451, Saudi Arabia; 4 Department of Engineering Mathematics and Physics, Faculty of Engineering, Mansoura University, El-Mansoura 35516, Egypt; 5 Department of Mathematics and Physics Engineering, Faculty of Engineering at Mataria, Helwan University, Cairo 11718, Egypt

**Keywords:** nanofibers, hydrogen production, energy application, electrospinning

## Abstract

In this study, we report on the fabrication and utilization of NiCr alloy nanoparticles (NPs)-decorated carbon nanofibers (CNFs) as efficient and competent non-precious catalysts for the hydrolytic dehydrogenation of ammonia borane (AB) at 25 ± 2 °C. The introduced NFs have been fabricated in one step using a high-temperature thermal decomposition of the prepared electrospun nanofiber mats (nickel acetate tetrahydrate, chromium acetate dimer, and polyvinyl alcohol) in an inert atmosphere. The chemical composition of the NFs with different proportions of Ni_1−x_Cr_x_ (x = 0.0, 0.1, 0.15, 0.2, 0.25, 0.3) was established via standard characterization techniques. These techniques proved the formation of disorder Cr_2_Ni_3_ alloy and carbon for all the formulations. The as-synthesized composite NFs exhibited a higher catalytic performance for AB dehydrogenation than that of Cr-free Ni–CNFs. Among all the formulations, the sample composed of 15% Cr shows the best catalytic performance, as more H_2_ was released in less time. Furthermore, it shows good stability, as it is recyclable with little decline in the catalytic activity after six cycles. It also demonstrates the activation energy, entropy (ΔS), and enthalpy (ΔH) with 37.6 kJ/mole, 0.094 kJ/mole, and 35.03 kJ/mole, respectively. Accordingly, the introduced catalyst has a lower price with higher performance encouraging a practical sustainable H_2_ energy application from the chemical hydrogen storage materials.

## 1. Introduction

The fast growth of the global population associated with social development and technologies requires a sufficient and sustainable energy supply. For this purpose, H_2_ has been adopted as one of the perfect substitute energy carriers. During storage, transit and utilization H_2_ causes safety problems. Thus, the storage of hydrogen in solid form overcomes these issues. Boron-hydrides (e.g., NaBH_4_, NH_3_BH_3_, LiBH_4_, etc.) are the best candidates for solid hydrogen storage as per the United States Department of Energy (US DOE) [1]. They have the potential for strengthening the hydrogen economy over time [2,3]. Their distinct features (e.g., high storage capacity (10–20 wt%) nonflammable, and nontoxic) make them a splendid alternative for the petroleum industry as fuel [4]. Ammonia boron (AB, NH_3_BH_3_) has high hydrogen storage capacity (19.6 wt%) with a low molecular weight (30.86 g mol^−1^) relative to other boron-hydrides. In the presence of a suitable catalyst, H_2_ can be produced from AB at room temperature to directly power the fuel cells for a cheaper and more convenient generation of electricity and hot water without additional efforts. A suitable, low cost, and efficient catalyst is considered the major obstacle for improving the kinetic properties under moderate conditions, and consequently the wide practical application of this system. A wide range of catalysts with different structures has been used in the hydrolysis process of AB. Noble metals (e.g., Pt, Ru, and Rh) are being used for the rapid production of hydrogen on account of having excellent durability, stability, performance, and tolerance against deactivation [5,6,7].

However, the higher cost and resource shortages limited their practical applications. Subsequently, the search for the recyclable and low-cost catalyst has become imperative for solving these issues. The cost-efficient first-row transition metals (e.g., cobalt (Co), nickel (Ni), Copper (Cu), and iron (Fe)) are available in abundance [8,9,10,11,12,13,14,15]. Among them, Ni NPs served as the most attractive and active catalysts in the catalytic hydrolysis of AB [9,16,17,18,19,20,21]. This is because of their environmental benignity, good performance, and room temperature ferromagnetic properties [22,23,24,25]. Moreover, Ni NPs tend to easily oxidize in air or aqueous solution; they also easily aggregate and fuse, owing to their magnetic-induced property and increased surface energy [9,19,22]. These issues not merely limit their catalytic activity level; they also sharply reduce the recycling performance. Several strategies have been investigated to stabilize Ni NPs and achieve higher H_2_ generation rates. These strategies include the supported Ni on the various matrices (e.g., foams, thin film, metal oxides, metal organic frameworks, etc.) [16,19,21,26,27,28,29,30,31,32,33]. Compared with the above supporters, nanocarbon materials provide a desirable surface to obtain a good attachment [34,35,36,37]. Furthermore, they have a good chemical stability, thermal stability, and adsorption capacity. Among the carbon nanostructures, CNFs have a higher surface area and more facile preparation compared to other nanocarbon materials. Many techniques have been used to produce nanofibers. Amongst these techniques, the simple electrospinning technique is considered the most suitable one due to its large production capacity at a low price [2,20]. The formed electrospun NFs containing a nanoporous structure facile the reactants entering and product leaving. Accordingly, CNFs easily adsorb the AB for contacting the catalyst surface and the facile releasing of H_2_. Our previous works exhibit that CNFs can assist with dispersing and stabilizing the transition metal NPs, and can be used for various applications (e.g., fuel cells and hydrogen production) [25,38].

Another strategy is the addition of an atomic diffusion barrier (e.g., Cr, Mo, and W) to Ni atoms [39]. It is considered an active route for enhancing the catalytic properties via inhibiting the agglomeration phenomena [39]. Li et al. reported that the addition of a small concentration of Cr or W to Co–B reveals a high hydrogenation activity for glucose [40]. Patel et al. [41] demonstrated that the addition of atomic diffusion barrier (e.g., Cr, Mo, and W) had a strong impact on the catalytic activity and performance of Co–B for sodium borohydride hydrolysis. In addition, Chen et al. [42] indicated that the Mo and W enhanced the activity and selectivity of Ni–B toward p-chloronitrobenzene hydrogenation. Recently, Kangkang Yang et al. [39] found that the addition of Cr, Mo, and W enhanced the catalytic activity of Ni NPs toward ammonia borane and hydrazine borane dehydrogenation.

Herein, we prepared bimetallic NiCr alloy NPs decorated within the surface of CNFs via an electrospinning technique for the first time. The prepared NFs showed excellent stability and good catalytic activity toward hydrogen generation from AB. Furthermore, NFs reveal a good stability.

## 2. Experimental Section

### 2.1. Chemicals Used

Polyvinyl alcohol (PVA) (MW = 65,000 g/mol) was obtained from the D C Chemical Company, Seoul, South Korea. Chromium acetate dimer (CrAc)**,** nickel acetate tetrahydrate (NiAc), and ammonia borane complex (AB) were from Sigma Aldrich, St. Louis, MO, USA. Deionized water (DI) was utilized as a solvent.

### 2.2. Preparation of Catalytic Nano-Fibers

Typically, 10 wt% of PVA solution was prepared by dissolving PVA granules in DI water at 50 °C with vigorously stirring. Stock solution (NiAc/PVA) was made by mixing the prepared NiAc and PVA aqueous solutions (weigh ration 1:3). An amount from CrAc aqueous solution was added to 20 mL of NiAc/PVA aqueous solution to obtain different solutions at the mixture-composed weight ratios of CrAc to NiAc 10 wt%, 15 wt%, 20 wt%, and 30 wt%, respectively. The final solutions were stirred at 60 °C for 6 h, and then cooled at room temperature for obtaining a clear sol–gel. The sol–gel was electrospun by using 22 kV of DC power supply with a tip-to-collector distance of 20 cm. Fabricated NFs were collected from a rotating cylinder wrapped with a polyethylene sheet. The obtained electrospun NF mats were dried overnight under a vacuum at 50 °C. Finally, the mats were sintered under an Ar atmosphere for 5 h at 800 °C with the heating rate of 3 °C/min.

### 2.3. Catalytic Hydrolysis of Ammonia Borane Complex

The catalytic performance of the prepared NFs in the AB dehydrogenation was conducted on a gas volumetric apparatus (water-filled gas burette system). The setup of the apparatus can be found elsewhere [4,14,15,20,43]. Typically, a reactor was set up containing 10 mL of aqueous solution and 100 mM (31.5 mg) of AB. The solution in the reactor was stirred by a Teflon-coated stirring bar in the presence of a determined amount of catalytic NFs. The evolved H_2_ was measured by the water replacement method. The rate of H_2_ evolved was studied at different catalyst concentrations to find the rate of the reaction. Furthermore, the activation energy involved in the catalytic hydrolysis was calculated by the catalytic performance at different temperatures. In order to change the temperature, the reactor was put on a bath that contained silicon oil with a temperature regulator. Also, the stability of NFs in the AB dehydrogenation at room temperature was studied. A controlled experiment was also conducted on the similar reaction conditions without any catalytic material. It was noted that no considerable hydrogen gas evolved in the controlled experiment.

### 2.4. Characterization Performed

The prepared catalysts were characterized using standard techniques such as A JEOL JSM-5900 scanning electron microscope (JEOL Ltd., Tokyo, Japan) and field-emission scanning electron microscope (FESEM, Hitachi S-7400, Tokyo, Japan), which were used to study the surface morphology of the fabricated catalysts. Powder X-ray diffraction (PXRD) (Rigaku Co., Tokyo, Japan) with Cu Kα (λ = 1.54056 Å) radiation was utilized to examine the phase and crystallinity of the prepared catalysts. A JEOL JEM-2200FS transmission electron microscope (TEM) operated at 200 kV and equipped with EDX (JEOL Ltd., Tokyo, Japan) was used to obtain high-resolution images [2,7].

## 3. Results

### 3.1. Physiochemical Characterization

**Scheme 1:**
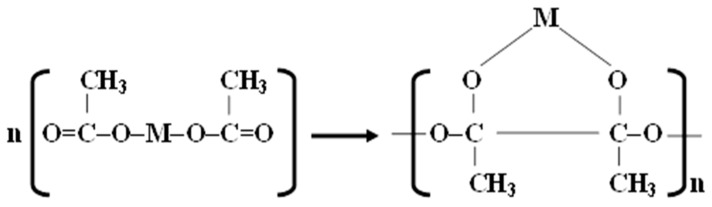
Polycondensation of the metal acetate precursors (M can be Ni or Cr).

Electrospinning is a powerful technique in the fabrication of inorganic NFs either from solutions or melts. The inorganic NFs fabrication process used a solution that consists of metal acetates or metal alkoxides as precursors. Polymers have been shown to have more simple polycondensation characteristics with metal acetates than those of alkoxides [2]. Furthermore, the electrospun solution that consisted of metal acetate showed an excellent morphology in the final product. The polycondensation process can be explained, as shown in Scheme 1.

The decomposition process of the precursor salts consist of metal acetates at high temperature under vacuum, and in the presence of an inert gas led to the generation of reducing gases (e.g., Co and H_2_), which result from the decomposition of acetate anions. The formed gases reduced the salt and metal oxides to form pure Ni and Cr and/or their alloys rather than their oxides [2]. The formation of Ni and Cr can be defined by the following reactions [44,45]:

Ni(CH_3_COO)_2_·4H_2_O → 0.86Ni(CH_3_COO)_2_ 0.14 Ni(OH)_2_ + 3.72 H_2_O + 0.28 CH_3_COOH (1)

0.86Ni (CH_3_COO)2 0.14 Ni(OH)_2_ → NiO + NiCO_3_ + H_2_O + CH_3_COCH_3_ (2)

NiCO_3_ → NiO + CO_2_ (3)

NiO + CO → Ni + CO_2_ (4)

(Cr(CH_3_COO)_2_)_2_.2H_2_O → 2Cr(OH)(CH_3_COO) + 2CH_3_COOH (5)

Cr(OH)(CH_3_COO) → 0.5 CrO + 0.5 CrCO_3_ + 0.5 H_2_O + 0.5 CH_3_COCH_3_ (6)

CrCO_3_ → CrO + CO_2_ (7)

CrO+CO → Cr + CO_2_ (8)

**Figure 1:**
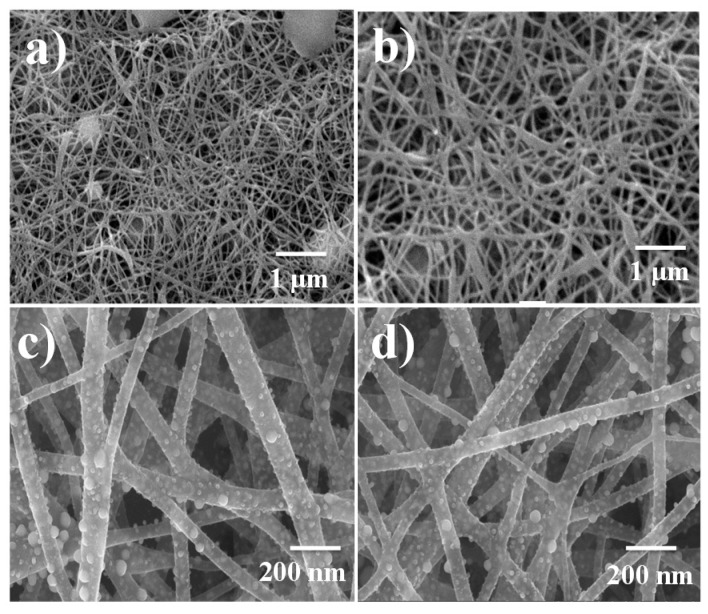
SEM image of electrospun NiAc/CrAc/PVA nanofiber (NF) mats after vacuum drying; (**a**) and (**b**), and field-emission scanning electron microscope (FESEM) image of the sintered powder sample; (**c**) and (**d**).

Various metal acetates have been applied to produce inorganic NFs with good morphology. Herein, the NiAc, CrAc, and PVA appear to have a good electrospun NF mats morphology, as shown in the SEM image (Figure 1a,b) after vacuum drying. The nanofibrous morphology of the sintered electrospun NF mats was not affected by the high calcination temperature. This may be attributed to the polycondensation of the NiAc and CrAc with PVA [3,20,44,46]. Figure 1c,d shows the sintered NFs obtained after the abnormal calcination of electrospun NF mats composed of NiAc/CrAc/PVA. It was observed in the figure that the NFs are cross-linked, and a net-like structure with some NPs is distributed on the rough surface. The formed fiber was decorated within the well-dispersed NPs.

### 3.2. Crystal Structure

**Figure 2:**
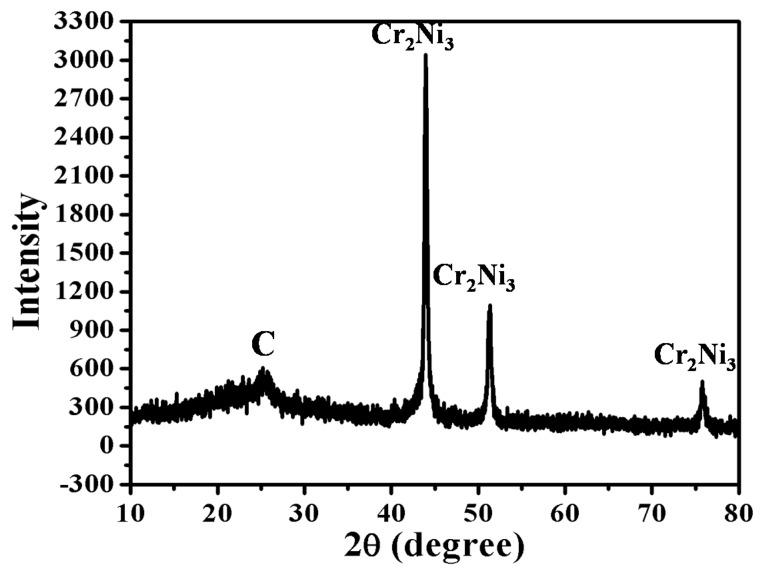
X-ray diffraction (XRD) pattern for the electrospun NiAc/CrAc/PVA NF mats after calcination at 800 °C in inert atmosphere.

The analytical analysis of the chemical composition of the produced NFs was achieved using an XRD technique (Figure 2). Nickel and chromium are in the same row and neighbors in the periodic table. They can form a solid solution until 30 wt% at room temperature, while above this value, an eutectic solution is formed. Accordingly, the identification peaks in the XRD database are overlapped. Moreover, the formed NiCr alloys have dissimilar crystal structures compared to the pure metals. For instance, nickel can be identified in the XRD pattern if peaks are detected at 2è values of 44.5°, 51.8°, and 76.4° corresponding to (111), (200), and (220) crystal planes, respectively (JCDPS #04-0850). However, chromium can be identified in the XRD pattern if peaks are detected at 2è values of 44.4°, 64.6°, and 81.72° matching to (110), (200), and (211) crystal planes, respectively (JCDPS #06-0694). Interestingly, the obtained 2è values at 43.9°, 51.2°, and 75.7° match well with (111), (200), and (220) of Cr_2_Ni_3_ (JCPDS # 65-629; Cr_2_Ni_3_) [47]. The same results were obtained for all formulations. According to the higher melting point of the two metals, 1455 °C and 1907 °C for Ni and Cr, respectively, than the used calcination temperature, vaporization of the produced bimetals is not predictable. Moreover, the nickel precursor represented a high amount in the initial electrospun solution compared to chromium; thus, the detected peaks in the XRD pattern (Figure 2) can be consigned to free Ni and a Cr_2_Ni_3_ alloy. It is worth noting that pure nickel and the bimetallic alloy can combine in the same nanoparticle, which was verified by the TEM EDX study below. Furthermore, a peak can be observed at 2θ ~26°, representing an experimental d spacing of 3.37, which confirmed the presence of graphite-like carbon (d (002), PDF#; 41-1487). According to our previous studies, Ni NPs and its alloys have vital roles in the catalytic transformation of polymer NFs into graphite-like carbon during the calcination process [2,46,48,49]. These results suggested a successful formation of Cr_2_Ni_3_ alloy NPs@CNFs.

### 3.3. Internal Structure

**Figure 3:**
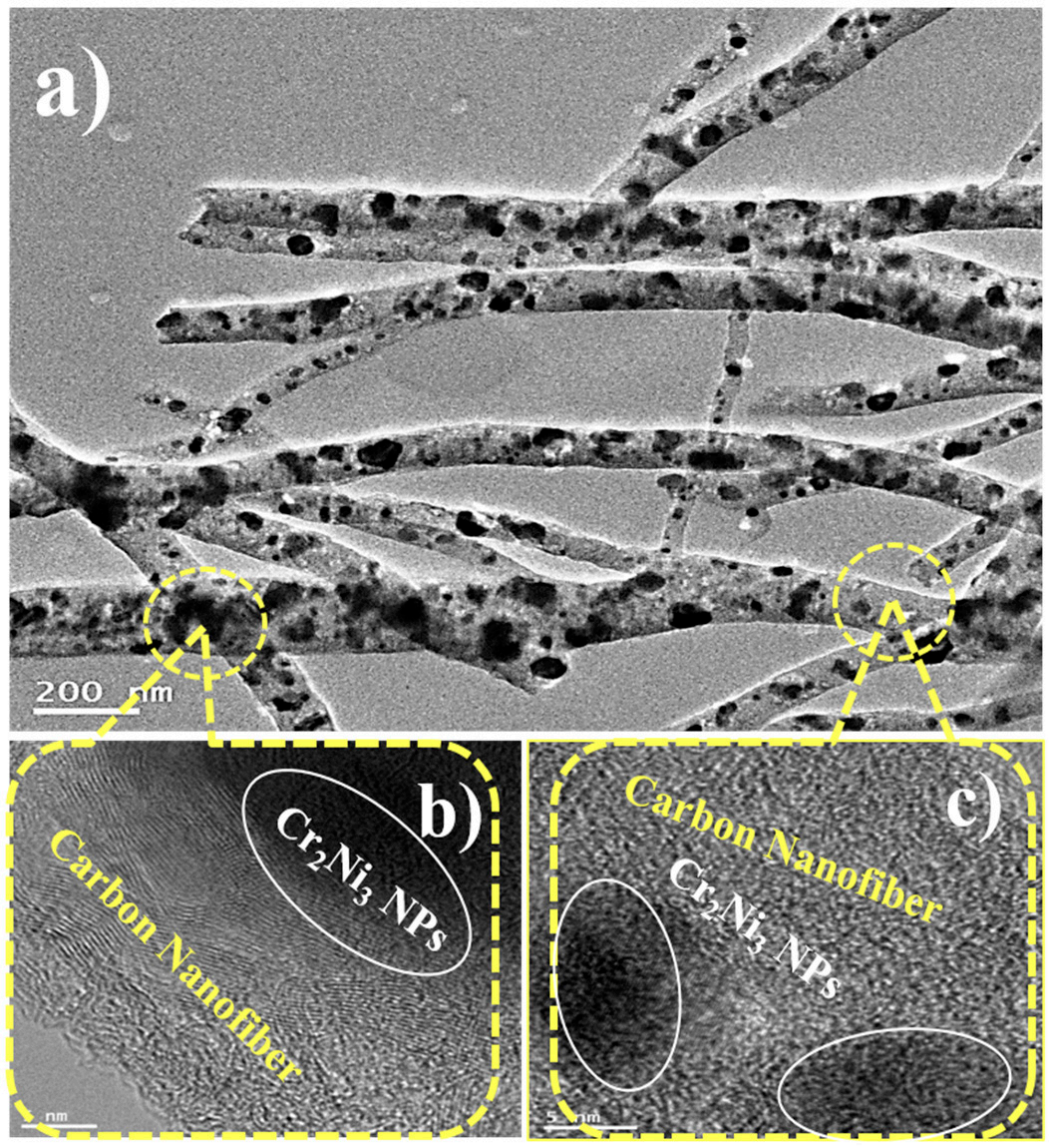
Normal TEM (**a**), and HRTEM images (**b**,**c**) of the as-synthesized Cr_2_Ni_3_ NPs@CNFs.

A typical TEM image of nanofibers (NFs) is presented in Figure 3a. It is clear that numerous dark spots from NPs are consistently anchored and distributed on the rough surface of NFs. HRTEM images are used to determine the crystalline of the produced NFs (Figure 3b,c). The clear appearance of the distance between the parallel crystal planes in Figure 3b,c affirmed that the NPs are crystalline, while the carbon is amorphous. The distance between two parallel crystals planes is mostly the same in the NPs in Figure 3b; this confirmed the presence of one phase only, as mentioned in the XRD analysis. Accordingly, the crystalline NPs and NFs matrix can be assigned to the detected Cr_2_Ni_3_ NPs and graphite, respectively.

**Figure 4:**
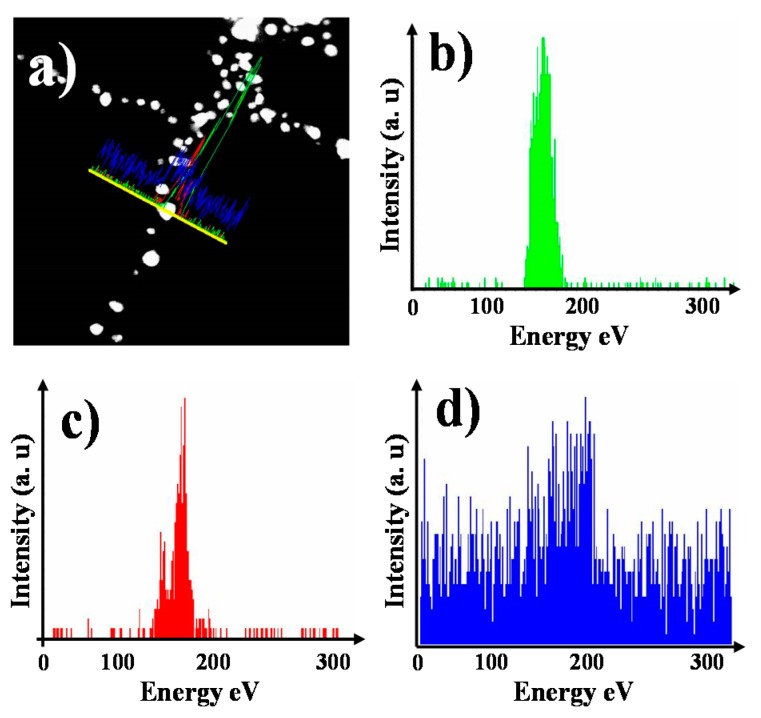
TEM EDX studies of Cr_2_Ni_3_ nanoparticles at carbon nanofibers (NPs@CNFs); STEM image, (**a**); Ni distribution, (**b**); Cr distribution, (**c**); and C distribution, (**d**).

To confirm the aforementioned result, the TEM-EDX was performed to check the elements and chemical composition of the obtained NFs. The STEM results (Figure 4a) show that the NPs are encapsulated inside the NFs matrix. TEM-EDX analysis corresponding to the line shown in Figure 4a, as shown in Figure 4b–d, indicates that the Ni and Cr nanoparticles (NPs) are uniformly distributed along the chosen line in the specified area, yielding the same distribution of Ni and Cr, which indicates the alloy formation. This verifies the XRD result regarding the formation of the Cr_2_Ni_3_ phase. Accordingly, the formed NPs and NFs were assigned to the Cr_2_Ni_3_ alloy and CNFs, respectively. Moreover, Cr_2_Ni_3_ NPs were packed well into a thin crystalline CNF layer. The layer was present at all points along the chosen line. Carbon could enhance the catalytic activity, material conductivity, and stability. Moreover, CNFs have high adsorptive capacity, which increases the attachment of the target with the catalytic material and easily creates an outlet product due to the porosity.

### 3.4. Hydrogen Release from Ammonia Borane Complex

**Figure 5:**
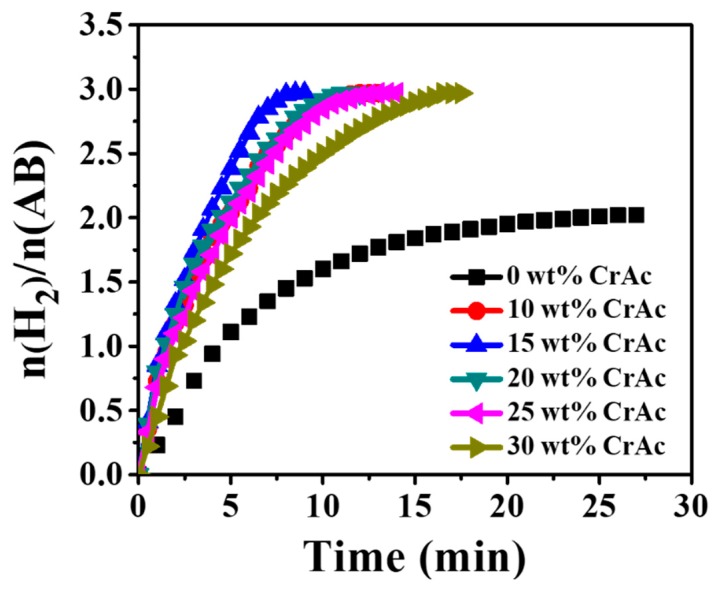
Evolution of H_2_ gas by the hydrolytic dehydrogenation of AB in the presence of Cr_2_Ni_3_ NPs@CNFs with different CrAc compositions.

The synthesized Ni_x_Cr_1-x_ NPs–CNFs were tested as catalysts for the AB dehydrogenation. Figure 5 shows plots of the time versus equivalent H_2_ (n_H2_ mol/n_AB_ mol) for the AB dehydrogenation in the presence of all the formulations and Ni–CNFs at the same molar ratio from the active catalyst and same concentration of AB at room temperature. As shown in the figure, the introduced NFs showed a higher catalytic performance for H_2_ generation compared to the pure metallic NPs (Ni–CNFs). This may be ascribed to the synergistic effect of the introduced alloy NPs. The pure metallic NPs took a relatively longer experimental time of 27 min to release 2 mol of H_2_/1 mol of AB. The hydrogen release rate increased and the duration time decreased as the amount of Cr increased, as the most of the reaction’s stoichiometric H_2_ was released. The sample composed of 15 wt% Cr took a shorter time to release stoichiometric H_2_ compared to the other formulations. In addition, it was observed in the figure that the H_2_ generation is directly proportional to the AB dehydrogenation duration time. This good performance can be attributed to the CNFs. The CNFs have a high absorptive capacity, a high surface area, and interact with a good dispersion of active catalysts, which creates a large number of active sites. It is well known that the electrospun CNFs have the well nanoporous structure, which facilitates the contact between the reactants and the active metals as well as H_2_ evolution. Accordingly, nanofibrous morphology introduced a high surface area and active sites for AB dehydrogenation. The performance can also be associated with the addition of Cr to Ni, which increases the surface area and works as an atomic barrier between Ni atoms for preventing agglomeration. Furthermore, the interaction between Ni and Cr species modified the electronic structure of the alloy [39].

Accordingly, the migration electrons from Cr to Ni enhance the active sites with the electron-enriched Ni NPs. These electrons enhance the detachment of H_2_ atoms from the catalyst surface. However, the increase of the Cr concentration and decrease of the H_2_ release could be due to the screening of the Ni active sites, which reduces the electron transfer. Furthermore, the decrease in the H_2_ generation at a Cr concentration lower than that of 15 wt% CrAc may be due to the surface oxidation.

**Figure 6:**
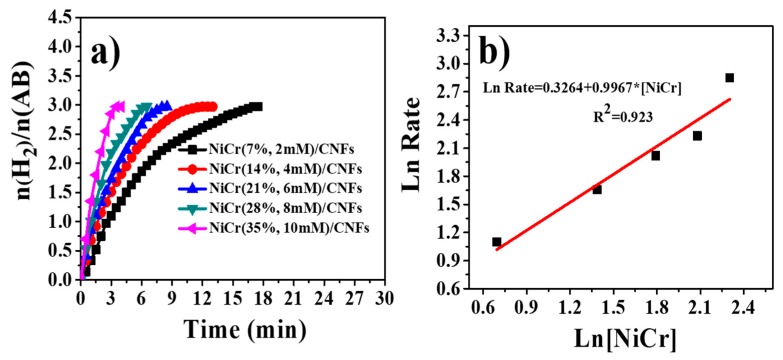
Effect of [Cr_2_Ni_3_] (15 wt% CrAc) on H_2_ gas generation from hydrolytic dehydrogenation of AB (100 mM) at 25 °C (**a**) and ln rate vs. ln [Cr_2_Ni_3_] (**b**).

Figure 6 demonstrates the graphs of (n H_2_/1 mole AB) versus the duration time of aqueous AB hydrolysis (31.5 mg and room temperature) in the presence of different concentrations of the introduced NFs (15 wt% sample). As shown in the figure, with an increase in the catalyst and the AB ratio, the time required for H_2_ generation decreases. From the linear portion of the different concentrations, a graph between the log H_2_ generation rates versus log catalyst concentration was plotted. From this graph, a straight line within a slope of 0.997 was obtained, indicating that the AB hydrolysis followed a pseudo-first-order reaction with respect to the concentration of the introduced NFs (Figure 6b).

**Figure 7:**
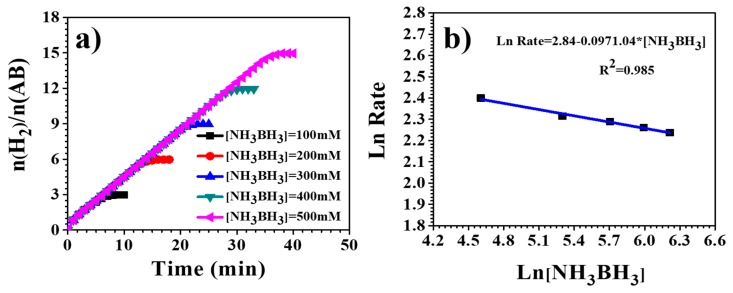
Effect of [NH_3_BH_3_] on H_2_ gas generation from the hydrolytic dehydrogenation of AB using Cr_2_Ni_3_ NPs@ CNFs (**a**) and ln Rate vs. ln [NH_3_BH_3_] (**b**).

The effect of AB concentration (100–500 mM) on the rate of H_2_ generation was also investigated. As shown in Figure 7, most of the stoichiometric hydrogen was evolved for each concentration. The logarithmic rate constant obtained from the linear portion of concentration (Figure 7b) was plotted versus logarithmic AB concentrations, and the slope was to be found to be −0.0979, demonstrating that the hydrolysis reaction followed the zero-order reaction with respect to the AB concentration.

**Figure 8:**
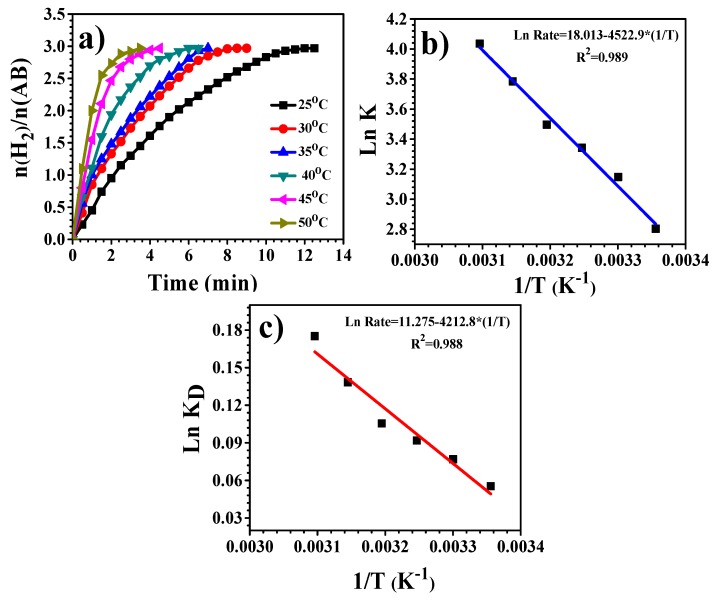
Evolution of H_2_ gas by the hydrolytic dehydrogenation of ammonia borane (AB) in the presence of Cr_2_Ni_3_ NPs@CNFs (15 wt% CrAc) at different temperatures (**a**), the plot of ln K vs. (1/T) (**b**) and the plot of ln K_D_ vs. (1/T) (**c**).

To determine the reaction activation energy (E_a_), activation entropy (ΔS), and activation enthalpy (ΔH), the hydrolytic reaction was carried out at different temperatures ranging from 298 K to 323 K. As shown in Figure 8, as expected, the duration time for H_2_ generation was reduced from 12.5 min to 3.5 min with an increasing chemical reaction temperature from 298 K to 323 K, respectively. The constant rate (k) value was determined from the linear portion from Figure 8a. The E_a_, ΔS, and ΔH of the reaction were determined from the Arrhenius and Eyring plots (Figure 8a,b), and were found to be 37.6, 0.094, and 35.03 kJ/mol, respectively. Here, the obtained E_a_ value is comparable to the Ni-based catalyst values reported in the literature (Table 1), indicating the superior catalytic performance of the introduced NFs (15 wt%, sample). In addition, the TOF was determined to evaluate the catalytic activity, and was found to be 5.78 mol H_2_ min^−1^ (mol metal^−1^). This value is also comparable to values reported in the literature for Ni-based catalysts (Table 1), indicating the high activities of the introduced NFs (15 wt%, sample).

**Figure 9:**
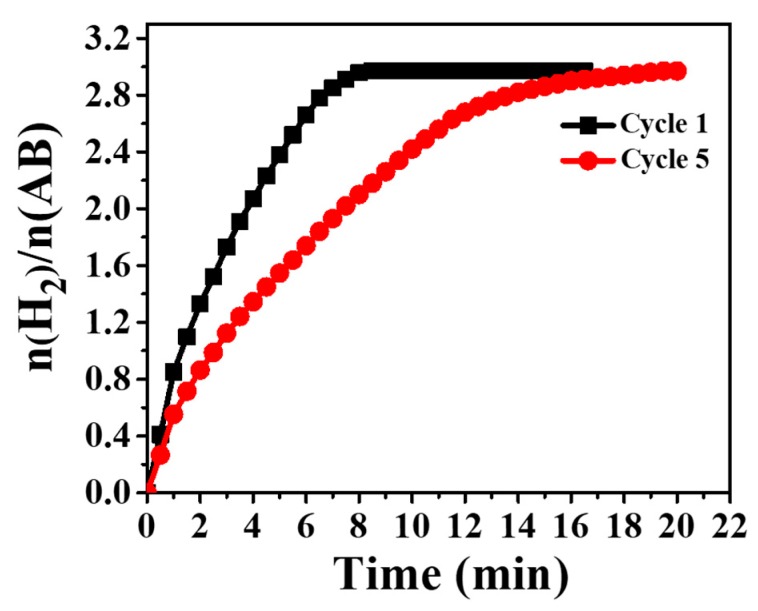
The catalytic performance of Cr_2_Ni_3_ NPs@CNFs (15 wt% CrAc at 25 °C) during recycling.

The reusability test is very significant for the practical applications of catalysts. In the present work, the reusability test of a 15 wt% CrAc sample was conducted for the hydrolytic reactions of AB. After the complete hydrolysis of AB (31.5 mg, 3n_H2_/1n_AB_), the catalyst was decanted from the solution via centrifugation, washed with deionized (DI) water, and then, the reactivate catalyst was again dispersed in the same solution with the addition of the same amount of AB for the next cycle. As shown in Figure 9, the catalytic activities of the catalyst indicate stability for two runs. However, a gradual decline was observed from the third cycle, reaching about 65% at the fifth cycle, demonstrating that the catalyst had a good reusability in the AB dehydrogenation at room temperature. The decline of catalytic activity may be attributed to the following: (1) the partial clustering of the metal NPs, (2) the increase of the solution viscosity, and (3) the passivation of the NFs’ surface, which was attributed to the accumulate boron products (e.g., metaborate) on the reaction solution and catalyst surface that inhibited the metal active sites [39,49].

## 4. Conclusions

In summary, we successfully prepared metallic Cr_2_Ni_3_ alloy nanoparticles-decorated carbon nanofibers via the abnormal sintering of electrospun nanofiber mats composed of nickel acetate tetrahydrate, chromium acetate monohydrate, and poly(vinyl alcohol) in the argon atmosphere. The synthesized nanofibers exhibited excellent catalytic activity for the hydrolytic dehydrogenation of AB compared to the Cr-free nanofibers. The sample content 15 wt% CrAc reveals the best catalytic performance among other formulations due to its lower duration time on the H_2_ generation from an aqueous solution of AB. The selected alloy shows a lower TOF and activation energy as compared to those available in the literature. The introduced catalytic NFs show a good stability in the hydrolytic dehydrogenation of AB. Accordingly, the optimum time was 3.5 min at 323 K to obtain 3 mole H_2_ using 15 wt% CrAc.

## Figures and Tables

**Table 1:** Comparison of activation energies and TOFs of different Ni-based catalysts for the hydrolytic dehydrogenation of AB.

Catalyst	E_a_ (KJ/mol)	TOF (mol_H2_. Mol_metal_^−1^. min^−1^)	References
Ni NPs	41.5	8.8	[17]
Ni/ZIF-8	41.5-	14.2	[28]
Fe–Ni alloy	41.5-	10.7	[12]
CoNi/graphene	13.49	16.4	[50]
Cu_0.64_Ni_0.36_/TiO_2_ nanotubes	36.14	15.9	[51]
Ni/BN	61.1	1.03	[52]
NiP/rGO	34.7	13.3	[22]
CuNi–MIL–101	32.2	20.9 (Cu)	[53]
Cu_0.2_Ni_0.8_/MCM41	38	10.7	[23]
CuNi@carbon	28.9	0.2	[20]
Cr_2_Ni_3_@carbon	37.6	5.78	This study
